# Patient with composite haemangioendothelioma containing angiosarcoma-like areas in the setting of congenital lymphoedema mimicking Stewart-Treves syndrome: a case report

**DOI:** 10.1186/s13000-023-01365-1

**Published:** 2023-06-22

**Authors:** Jan Balko, Andrej Ozaniak, Lenka Krskova, Zuzana Strizova, Robert Lischke, Josef Zamecnik

**Affiliations:** 1 Department of Pathology and Molecular Medicine, 2nd Faculty of Medicine, Charles University and Motol University Hospital, Prague, Czech Republic; 2 3rd Department of Surgery, 1st Faculty of Medicine, Charles University and Motol University Hospital, Prague, Czech Republic; 3 Department of Immunology, 2nd Faculty of Medicine, Charles University and Motol University Hospital, Prague, Czech Republic

**Keywords:** Composite haemangioendothelioma, Angiosarcoma-like areas, Stewart-Treves syndrome, Congenital lymphoedema, Angiosarcoma, Vascular neoplasm, Case report

## Abstract

**Background:**

Composite haemangioendothelioma is a rare vascular neoplasm with indolent to intermediate malignant potential. Diagnosis of this disease relays on histopathological identification of at least two different morphologically distinctive vascular components in proper clinical settings. Exceedingly rare cases of this neoplasm can exhibit areas resembling high-grade angiosarcoma, which does not change the biological behaviour. Such lesions tend to occur in the setting of chronic lymphoedema and thus, can mimic Stewart-Treves syndrome, which has a much worse clinical outcome and prognosis.

**Case presentation:**

We present a case of 49 years old male suffering from chronic lymphoedema of the left lower extremity who had developed a composite haemangioendothelioma with high grade angiosarcoma-like areas mimicking the Stewart-Treves syndrome. Given the multifocality of the disease, the only potentially curable surgical treatment would be hemipelvectomy, which was refused by the patient. The patient has been followed-up, with no signs of local progression of the remaining disease, nor a distant spread outside the involved extremity for two years.

**Conclusions:**

Composite haemangioendothelioma represents a rare malignant vascular tumour, with significantly more favourable biological behaviour than angiosarcoma, even in cases where angiosarcoma-like areas are present. For that reason, composite haemangioendothelioma can be easily misdiagnosed as true angiosarcoma. The rarity of this disease unfortunately hampers the development of clinical practice guidelines and the implementation of treatment recommendations. Most of the patients with localized tumour are treated by wide surgical resection, without neo- or adjuvant radiotherapy or chemotherapy. However, in the case of this diagnosis, the watch-and-wait approach is better than mutilating procedure, highlighting the necessity of establishing of the correct diagnosis.

## Background

Composite haemangioendothelioma (CHE) is a rare vascular neoplasm, currently described as a borderline tumour or a tumour with intermediate malignant potential. This description reflects large uncertainty in its clinical behaviour and patient prognosis. Local aggressiveness and recurrence are well documented, but metastatic behaviour has been rarely reported [1–3]. The tumour itself represents a mixture of morphologically heterogeneous vascular neoplasm [1, 4]. Dermis and subcutis of the extremities are the most common sites of occurrence, but multiple other sites can also be affected, including visceral ones [1, 5–8].The previously published cases of CHE implicate, that this disease can be found in many locations and forms multinodular masses [7–15]. It occurs at any age, but the majority of cases tend to appear during the forth to fifth decades of life with a slight female predominance. Risk factors for developing CHE include chronic lymphoedema, vascular malformations, Maffucci syndrome and irradiation [6]. The lack of clinical experience and rarity of this disease is mirrored by non-existing standards of therapeutic care.

Diagnosis of CHE relays on histopathological findings. Minimal diagnostic criteria for CHE include the presence of at least two morphologically different vascular components of the tumour [5]. Variable admixture of benign, intermediate and/or malignant vascular tumours can be observed. The most common findings are represented by a combination of retiform haemangioendothelioma, epitheloid haemangioendothelioma and/or Dabska type haemangioendothelioma/papillary intralymphatic angioendothelioma (PILA) [1, 4]. Other possible described distinct vascular components include kaposiform haemangioendothelioma, epitheloid/spindle cell/capillary/cavernous haemangioma, lymphangioma and angiomatosis [3, 4]. If the tumour arises in the setting of pre-existing vascular malformation, the identification of two additional components is required along to the (usually lymphatic) malformation itself.

Approximately half of the cases of CHE contain areas resembling angiosarcoma, which is usually well-differentiated [1, 2, 16]. However, high-grade (HG) angiosarcoma-like areas have also been described and recognized by WHO in exceedingly rare cases, sometimes even with epithelioid morphology [4]. In such cases the diagnosis of conventional angiosarcoma must be excluded on the clinical and morphological ground to support the proposed diagnosis of CHE. These requests include morphological identification of at least one area of other non-angiosarcomatous vascular component based on histopathological examination and clinical follow-up without any evidence of rapid progression or spread of the disease.

Chronic lymphoedema is well-known risk factor for the development of several vascular neoplasms including HG angiosarcoma [17]. The occurrence of (lymph)angiosarcoma in the terrain of lymphoedema is called the Stewart-Treves syndrome. Lymphoedema in this condition is usually attributed to post-mastectomy, but can be of any other origin [18]. In contrast to CHE with HG angiosarcoma-like areas, which still demonstrate indolent behaviour, the Stewart-Treves syndrome tends to affect older patients and commonly leads to early metastatic spread of the disease resulting in a very poor prognosis [19–22]. The essential prognostic difference underlines the importance of distinguishing between these two entities [23].

## Case presentation

We present a case of 49 years old Caucasian male suffering from congenital lymphoedema of the left lower extremity who developed composite haemangioendothelioma during his adulthood. Because of the lymphoedema, the patient was first operated on at the age of 6 years, which had only a minor effect on overall condition of the patient. Later on in his life, he experienced multiple erysipelas and at the age of 47 a vascular tumour was detected in the setting of the lymphoedema as its complication. In another institution, a microscopically non-radical (R1) resection was performed and a diagnosis of angiosarcoma was concluded based on the histopathological evaluation of the tumour. The patient was not indicated for any adjuvant therapy, or re-resection. He was followed up and the first control PET/CT scan showed neither local relapse nor distant spread. Local progression was detected after one year of follow up and the patient was re-operated. Second R1 resection was performed and subsequent histopathological examination revealed an angiosarcoma again. This diagnosis was supported by an early second local relapse after a month. After that, the patient was reported to our sarcoma centre for further treatment.

**Fig. 1:**
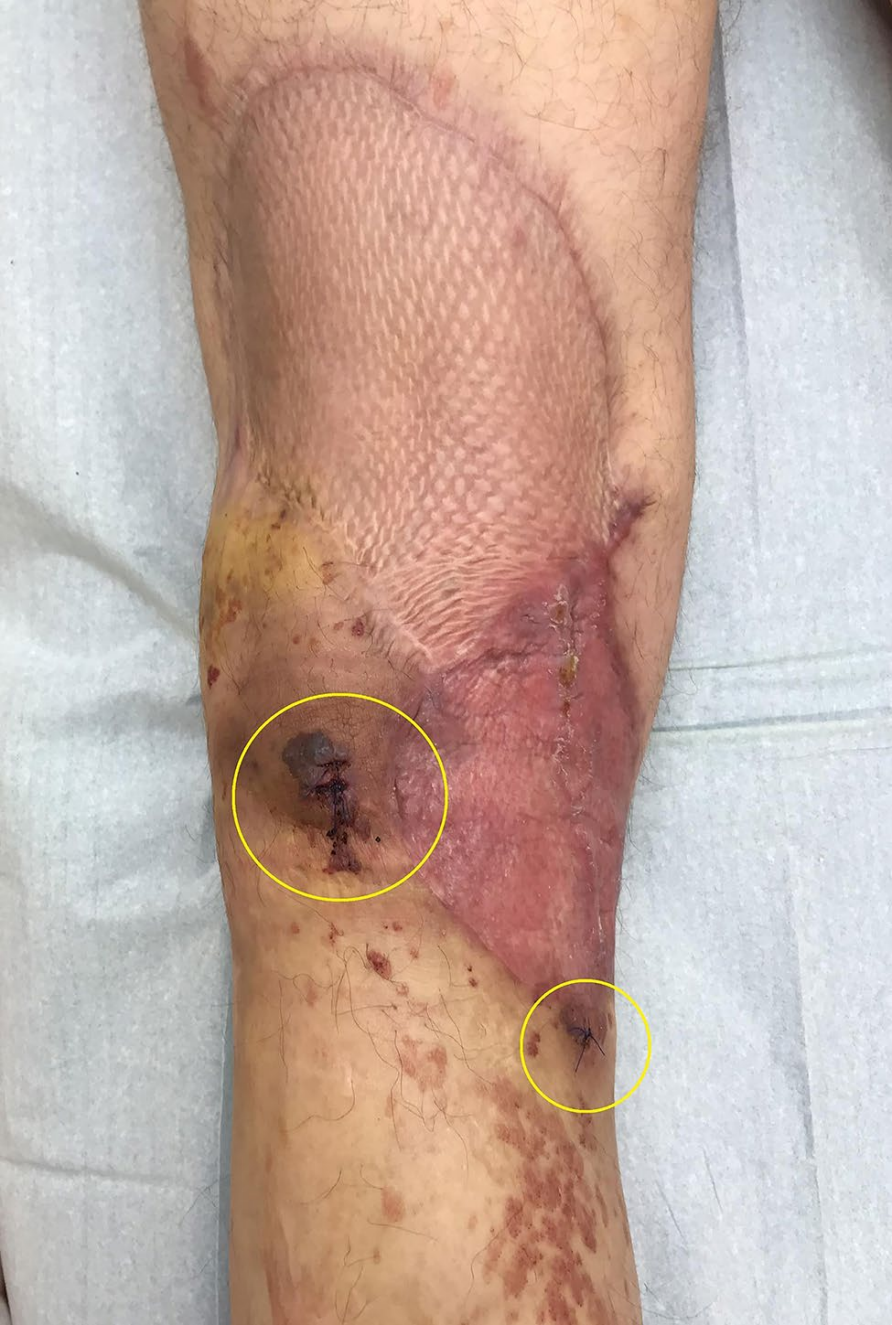
Clinical investigation of the skin - two purple skin affections in the popliteal area at the edges of dermo-epidermal mesh skin graft

**Fig. 2:**
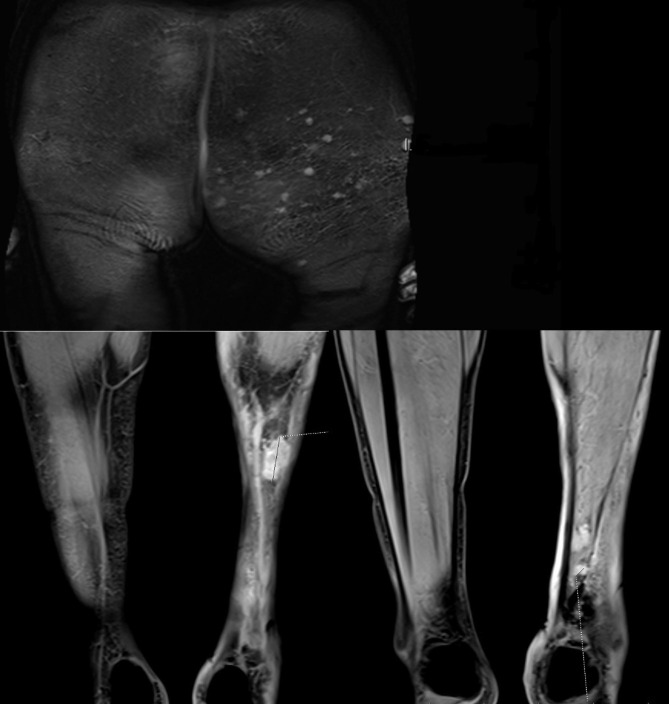
MRI of the lower extremities - multiple small subcutaneous nodules located in the left gluteal area, left calf and left hamstring area

**Fig. 3:**
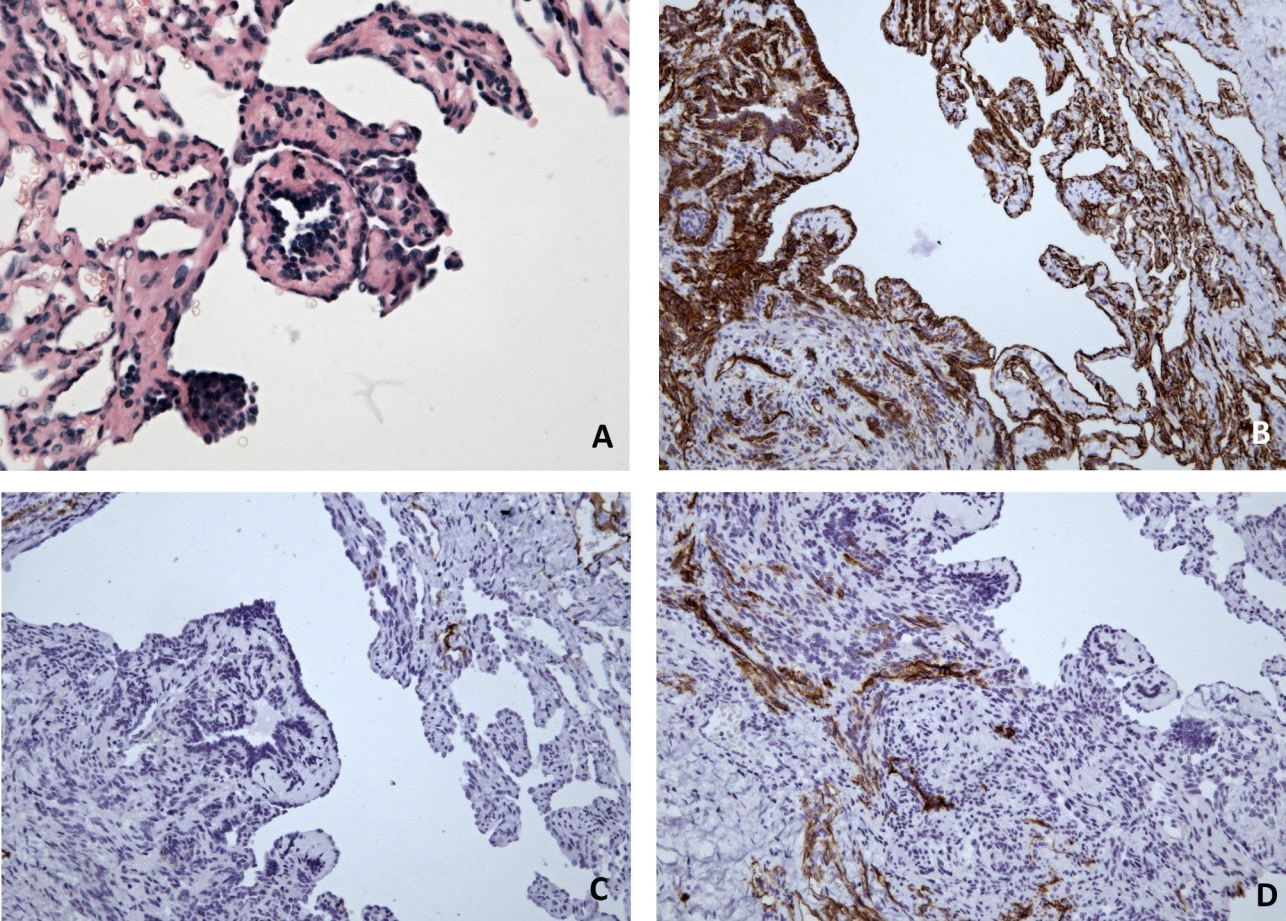
Light microscopy of Dabska-like component of the composite hemangioendothelioma - tumour consisted of cavernous vascular spaces containing papillary protrusions with hyalinised cores, which were covered with columnar endothelial cells with hobnail or even matchstick-like features. There were neither mitoses nor severe nuclear atypia present. (**a** H&E staining, 400x) Immunohistochemistry showed diffuse positivity for CD31 of the tumour cells (**b** IHC, 200x), while CD34 staining was negative (**c** IHC, 200x). Focal D2-40 positivity within slit-like spaces at the periphery was observed (**d** IHC, 200x)

During the clinical investigation, two purple skin affections in the popliteal area at the edges of the dermo-epidermal mesh skin graft were found (Fig. 1). The left lower extremity showed chronic disfiguration as a result of multiple erysipelas and chronic lymphoedema. Scattered ecchymoses were also present. In the left gluteal area, multiple small nonspecific purple skin affections were visible. For staging purposes, whole-body PET/MR was performed and revealed multiple small subcutaneous nodules located in the left gluteal area, left calf and left hamstring area (Fig. 2). Every nodule demonstrated low metabolic activity; however, the finding raised clinical suspicion for metastases of the previously diagnosed angiosarcoma. During the multidisciplinary tumour board, a bioptic confirmation of the process within gluteal space was indicated. Under local anaesthesia excision biopsy of the skin affection in the gluteal area was performed and sent for histopathological analysis. Within dermis, there was a cavernous vascular tumour (diameter 5 mm) detected, which consisted of dilated thin-walled lymphatic vessels containing papillary protrusions at the periphery. The papillary protrusions contained hyalinised cores in the centre and were covered with columnar endothelial cells with hobnail or even matchstick-like features. There were present neither mitoses nor severe nuclear atypia. Immunohistochemistry (IHC) showed diffuse positivity of the neoplastic cells for CD31, ERG and FLI-1. Tumour showed also focal D2-40 positivity within slit-like spaces at the periphery. IHC markers CD34, HHV-8, CK-KL1 and smooth muscle actin were negative. Based on the clinical background (localization within the dermis of the gluteal area), morphology and immunophenotype of tumorous cells, the suspicion for papillary intralymphatic angioendothelioma (PILA) was raised in contrast to the previous diagnosis (Fig. 3). The expected structures of angiosarcoma were not identified within the sample.

**Fig. 4:**
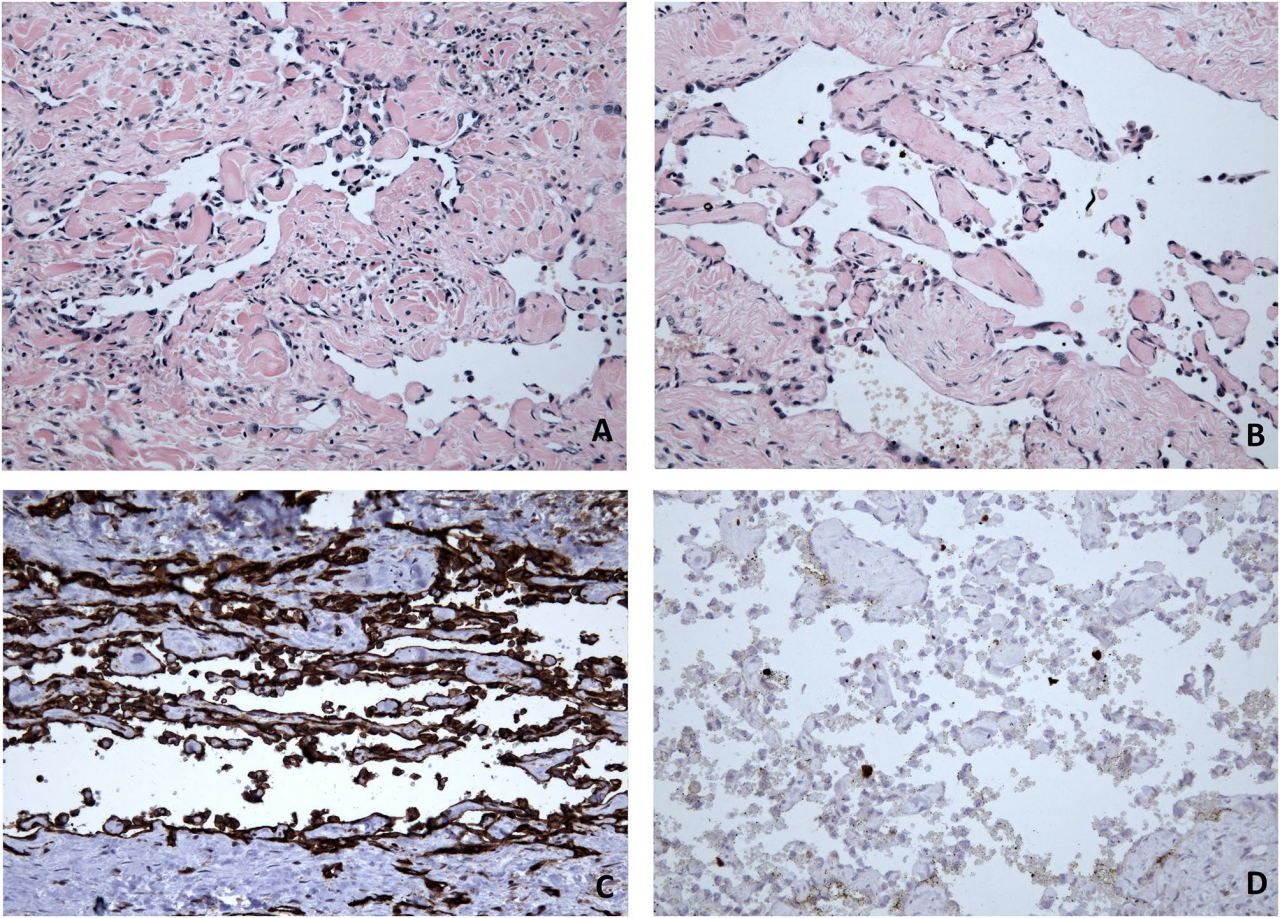
Light microscopy of retiform hemangioendothelioma-like component of the composite hemangioendothelioma - dermal based vascular tumour with retiform arrangement resembling rete testis, which consisted of branching vascular channels lined with hobnail-shaped bland endothelial cells (**a** H&E staining, 200x). At some portions, papillary protrusions with hyalinised cores were found (**b** H&E staining, 200x). Immunohistochemistry showed diffuse CD31 positivity (**c** IHC, 200x), while CD34 was negative (not shown). Proliferation marker Ki-67 was low with positivity of isolated cells (**d** IHC, 200x)

To evaluate the uncertain undergoing process, the next multidisciplinary meeting led to an indication for excision of the recurrent focus and bioptic evaluation of the nodules of the calf and gluteal area. The operation was performed in February 2022 in form of an excision of two skin affections of the popliteal area (diameter 43 and 78 mm) and another biopsy of the tumour in the calf and gluteal area (diameter 20 mm and 10 mm). Histopathological examinations showed a new finding (Fig. 4) – the dermal-based vascular tumour with the retiform arrangement was detected, which consisted of branching vascular channels lined with hobnail-shaped bland endothelial cells. At some portions, papillary protrusions with hyalinised cores were found. There were neither mitoses nor nuclear atypia again. On the other hand, no association with the lymphatic vessels was found this time. IHC showed diffuse CD31 positivity and CD34 negativity of the neoplastic cells. Proliferation marker Ki-67 was low with positivity in approximately 10% of the cells within hot spots. Even though the histopathological finding was similar to the previous sample, due to the multifocality of the process, arborizing retiform architecture of the tumour and lacking association with lymphatic vessels in the current biopsy, the diagnosis of retiform haemangioendothelioma was made.

**Fig. 5:**
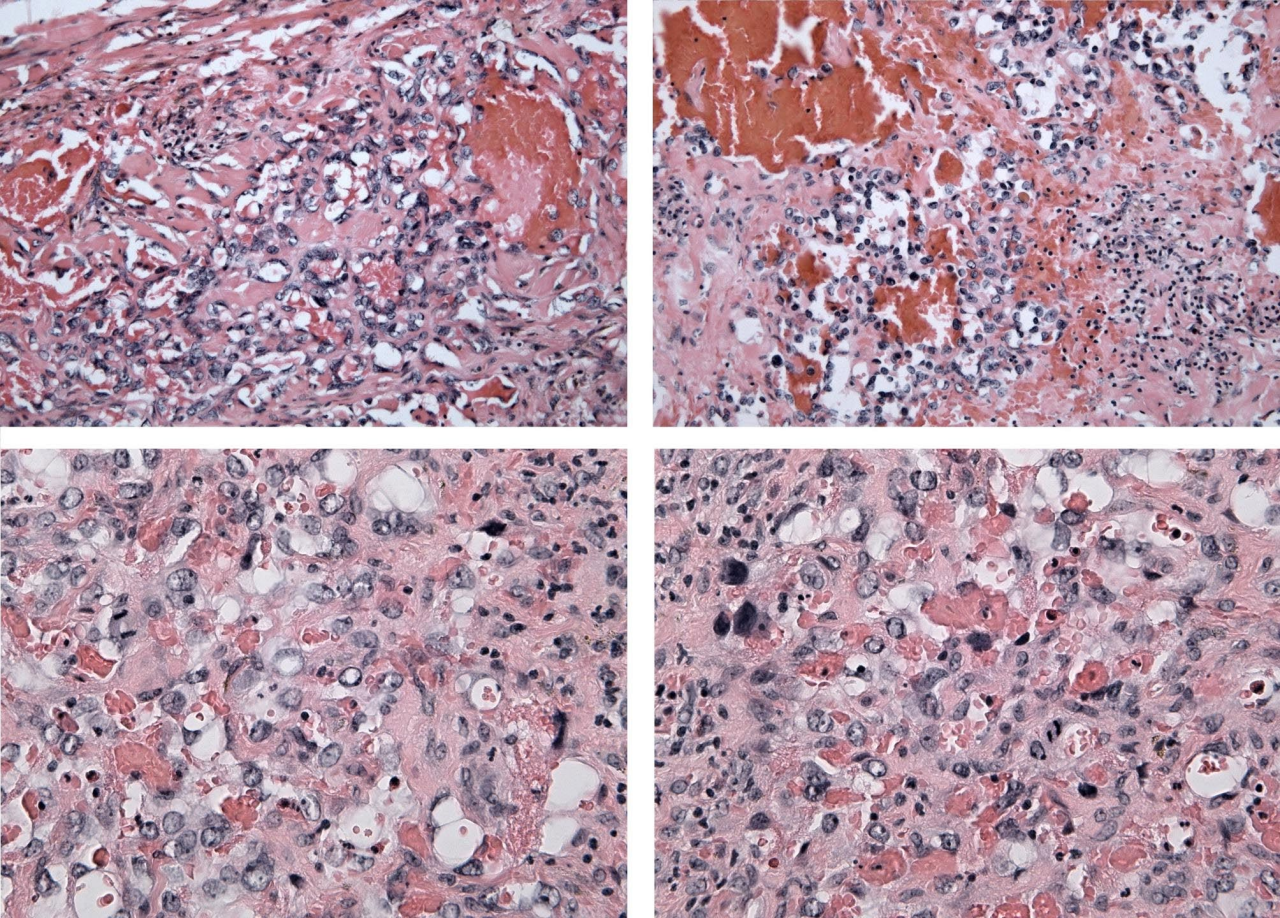
Light microscopy of high grade angiosarcoma-like component of the composite hemangioendothelioma - infiltrative vascular tumour consisting of erythrocytes filled slit-like spaced dissecting the dermis and more-solid areas with residual lumen formation. The neoplastic cells show high grade atypia with large vesicular nuclei and prominent nucleoli. Bizarre nuclei are also present. Numerous mitotic figures and chronic inflammation can be found (H&E staining, 200-400x)

Because of the absence of angiosarcomatous structures, the original bioptic material was obtained from the first resection for the second opinion. But, the second look examination confirmed the former diagnosis of HG angiosarcoma: at the border between dermis and sub-cutis there was an infiltration of solid tumour consisting of elongated oval to spindle shaped cells showing marked nuclear atypia and numerous mitoses, including atypical forms. Erythrocytes filled slit-like spaced dissecting the solid areas were detected. IHC showed a similar immune profile of the neoplastic cells (CD31 positive; CD34 and D2-40 negative) and significantly higher proliferation (40–50%) based on the Ki-67 proliferation marker. Such a finding was consistent with the original diagnosis of HG angiosarcoma (Fig. 5). However, at the base of the sample, small areas with a morphology of retiform haemangoendothelioma similar to the previous finding were spotted.

Based on the histopathological findings of three different vascular components resembling PILA, retiform haemangioendothelioma and HG angiosarcoma, the final diagnosis of CHE with HG angiosarcoma-like areas was established.

Recently it has been discovered that CHE can harbour a fusion gene *YAP1::MAML2*, especially among children with acral localisation of the tumour [24]. We performed next-generation sequencing (NGS) to investigate this gene fusion and to exclude other entities which could mimic such lesion. Using Fusion Plex Sarcoma V2 panel (Archer) the fusions of the following genes were excluded: *ALK, BCOR, BRAF, CAMTA1, CCNB3, CIC, CSF1, EGFR, EPC1, ERG, ESR1, ETV1, ETV4, ETV5, ETV6, EWSR1, FGFR1, FGFR2, FGFR3, FOS, FOSB, FOXO1, FUS, GLI1, HMGA2, JAZF1, MBTD1, MDM2, MEAF6, MET, MGEA5, MKL2, NCOA1, NCOA2, NCOA3, NR4A3, NTRK1, NTRK2, NTRK3, NUTM1, PAX3, PDGFB, PDGFRA, PHF1, PLAG1, PRKCA, PRKCD, RAF1, RET, ROS1, SS18, STAT6, TAF15, TCF12, TFE3, TFG, USP6, VGLL2, YAP1, YWHAE*).

In conclusion no gene fusion was identified, including *YAP1::MAML2*. However this finding does not exclude the diagnosis of CHE as for such gene fusion usually does not occur among adult patients outside acral localisations [24]. Moreover, we also examined *MYC* amplification using fluorescent in situ hybridisation (FISH) as there is an evidence in literature that high-level *MYC* gene amplifications (at 8q24.21) occur in majority of post-irradiation and chronic lymphoedema-associated angiosarcomas [25]. However *MYC* amplification was not found in this case, which further supports the diagnosis of CHE favouring it over angiosarcoma.

Even though CHE usually shows indolent behaviour, the multifocality of the process in the setting of chronic lymphoedema allowed radical surgical treatment, which would have to be hemipelvectomy in this case. Such a procedure was rejected by the patient. Therefore, the current course of action is the follow up with periodical PET/MR scans. In case of local progression of any nodule, the patient would be indicated for the extirpation of the lesion. However, no growth of the remaining lesions was detected in the follow-up so far and there has been no metastatic spread of the disease since the first detection of the tumour two years ago.

## Discussion and conclusions

Stewart-Treves syndrome first described by Drs. Steward and Treves, represents a development of (lymph)angiosarcoma in the terrain of chronic lymphoedema, usually after mastectomy [18]. Nevertheless, conditions such as chronic lymphoedema, chronic venous stasis and chronic infections are well-known risk factors for the development of other vascular neoplasms, such as retiform haemangioendothelioma or CHE [6].

CHE is an extremely rare histological entity, with a potentially fatal impact on patient. It has an intermediate malignant potential and therefore it can lead to local recurrences, loco-regional or rarely to distant spread [1]. Predominantly it occurs in the extremities, but multiple other sites can also be affected [1, 5–8]. Single case reports describe many other various localizations such as head and neck, oral cavity, hypopharynx, stomach, liver, spleen, kidney, pericardium and heart [7–15]. It can occur at any age, but forth to fifth decades are the most common. The usual clinical presentation represents single or multiple purple to red papules or nodules, sometimes sized up to 30 cm [2]. Diagnosis of this disease relays on histopathological identification of at least two different morphologically distinctive vascular components in proper clinical settings [5]. Exceedingly rare cases of this neoplasm can exhibit areas resembling high grade angiosarcoma, as it was observed in our case [4].

In this case report, the final diagnosis of CHE with HG angiosarcoma-like areas was established based on histopathological analysis of three different vascular lesions resembling PILA, retiform haemangioendothelioma and HG angiosarcoma. The epitheloid haemangioendothelioma, PILA and retiform haemangioendothelioma are described as the most common histopathological components of CHE in the literature [1, 4]. Clinical features such as age, affection of the dermis and subcutis of the lower extremity and the setting of chronic lymphoedema also support the diagnosis of CHE [1, 5, 6]. Moreover, IHC revealed the usual immunophenotype of CHE (diffuse positivity of CD31, ERG and FLI-1; focal positivity of D2-40; CD34 and HHV-8 negative) and NGS excluded many other lesions in the differential diagnostic spectrum on the molecular level. FISH analysis also confirmed that the tumour cells do not harbour *MYC* amplification, which occurs in majority of post-irradiation and chronic lymphoedema-associated angiosarcomas [25], supporting the diagnosis of CHE. Among others, this case report highlights the importance of proper sampling of bioptic material and shows that needle biopsies may not be sufficient as they do not commonly yield enough components of the tumour required for proper diagnostics of this rare disease [4].

The most important feature distinguishing between CHE with HG angiosarcoma-like areas and the Stewart-Treves syndrome is the biological behaviour of the tumour [23]. During the two years of clinical follow-up, our patient did not develop a distant spread of the disease. Such finding corresponds to the typically indolent behaviour of CHE, which can lead to multiple local recurrences and late regional lymph node metastases; the distant metastases are exceptionally rare [4]. Moreover, only a few cases of tumour-related death have been reported [26]. In contrast, angiosarcoma appears to be a highly aggressive disease, even if well-differentiated [1, 2]. More than half of patients die of the disease within one year, often with distant metastatic spread of the tumour [23]. Among other factors associated with poor outcomes of angiosarcoma is the size of the tumour, which was significantly large in our patient.

Currently, we are lacking the standards of care for patients with this disease. Most of the published cases describe angiosarcomas in general or angiosarcomas as a part of Stewart-Treves syndrome. We were unable to find any suggested treatment regimens for CHE with HG angiosarcoma-like areas developed in the setting of chronic lymphoedema. Most patients with localized disease, in previously published articles, were treated by surgery alone. The disease itself shows potentially favourable biological behaviour compared to conventional angiosarcoma [23]. However, even after microscopic radical wide resection, local recurrence was observed in 32% and metastatic potential in 11.5% of patients in a study by McNab et al. [6]. In a study by Perry et al., eleven patients with expression of neuroendocrine markers had even worse results [27].

The high rate of local recurrence is an argument for multimodal therapy. The systemic treatment used in angiosarcomas includes regimes based on anthracyclines [28, 29]. Taxanes and gemcitabine regimes represent another possible option of systemic treatment [30, 31]. In general, the response rate to treatment is low and the potential adverse effects are relatively high. Recently, immunotherapy with checkpoint inhibitors had been included as a potential second line of treatment [32]. Therefore, in patients with advanced, but stable composite haemangioendothelioma, it is not clear whether to push aggressive systemic treatment or to apply watch-and-wait approach only.

In conclusion, composite haemangioendothelioma represents a rare malignant vascular tumour, with significantly more favourable biological behaviour than angiosarcoma. If a radical treatment would require mutilating procedure and the disease is stable, we propose a watch-and-wait approach.

## Data Availability

All data generated or analysed in the current article are available from the corresponding author on reasonable request.

## Abbreviations

CHE: Composite haemangioendothelioma
FISH: Fluorescent in situ hybridisation
HG: High grade
PILA: Papillary intralymphatic angioendothelioma
PET/CT: Positron emission tomography computed tomography
PET/MR: Positron emission tomography magnetic resonance
IHC: Immunohistochemical staining
NGS: Next generation sequencing
